# Growth standard charts for monitoring bodyweight in dogs of different sizes

**DOI:** 10.1371/journal.pone.0182064

**Published:** 2017-09-05

**Authors:** Carina Salt, Penelope J. Morris, Alexander J. German, Derek Wilson, Elizabeth M. Lund, Tim J. Cole, Richard F. Butterwick

**Affiliations:** 1 WALTHAM Centre for Pet Nutrition, Mars Petcare, Waltham on the Wolds, Leicestershire, United Kingdom; 2 Institute of Ageing and Chronic Disease, University of Liverpool, Neston, Cheshire, United Kingdom; 3 Banfield Pet Hospital, Vancouver, WA, United States of America; 4 Institute of Child Health, University College London, London, United Kingdom; US Geological Survey, UNITED STATES

## Abstract

Limited information is available on what constitutes optimal growth in dogs. The primary aim of this study was to develop evidence-based growth standards for dogs, using retrospective analysis of bodyweight and age data from >6 million young dogs attending a large corporate network of primary care veterinary hospitals across the USA. Electronic medical records were used to generate bodyweight data from immature client-owned dogs, that were healthy and had remained in ideal body condition throughout the first 3 years of life. Growth centile curves were constructed using Generalised Additive Models for Location, Shape and Scale. Curves were displayed graphically as centile charts covering the age range 12 weeks to 2 years. Over 100 growth charts were modelled, specific to different combinations of breed, sex and neuter status. Neutering before 37 weeks was associated with a slight upward shift in growth trajectory, whilst neutering after 37 weeks was associated with a slight downward shift in growth trajectory. However, these shifts were small in comparison to inter-individual variability amongst dogs, suggesting that separate curves for neutered dogs were not needed. Five bodyweight categories were created to cover breeds up to 40kg, using both visual assessment and hierarchical cluster analysis of breed-specific growth curves. For 20/24 of the individual breed centile curves, agreement with curves for the corresponding bodyweight categories was good. For the remaining 4 breed curves, occasional deviation across centile lines was observed, but overall agreement was acceptable. This suggested that growth could be described using size categories rather than requiring curves for specific breeds. In the current study, a series of evidence-based growth standards have been developed to facilitate charting of bodyweight in healthy dogs. Additional studies are required to validate these standards and create a clinical tool for growth monitoring in pet dogs.

## Introduction

The growth phase is fundamental to the lifelong health and wellbeing of all humans. A growth pattern that deviates from optimal can result from malnutrition or the presence of an underlying developmental disorder. An atypical growth pattern can also predispose to obesity, with overly rapid growth or catch-up growth being known risk factors [1]. Growth standards, such as those created and promoted by the World Health Organisation [2,3], have become an essential component of the human paediatric tool kit, allowing trained health professionals to compare an individual's growth and development with that of a healthy reference population [2,3]. Such standards can also be used to describe the requirements, health, and wellbeing of populations as well as determining and monitoring the effectiveness of interventions or environmental factors [3,4].

As with humans, growth patterns that deviate from ideal can be indicative of either the presence of or potential for disease in companion animals [5]. Some early life diseases can be associated with retarded growth, whilst over-nutrition can be associated with becoming overweight [6] and with developmental musculoskeletal disorders (e.g. diseases of the osteochondrosis group) in predisposed breeds such as the Great Dane [7,8]. However, there is limited information and little current guidance available on what constitutes optimal growth in dogs, with growth standards equivalent to those used in children not currently available. Indeed, the canine species presents a unique challenge when attempting to develop such standards, because of the diversity of breeds with vastly different shapes and sizes, ranging from the 1 kg Chihuahua to the 115 kg St Bernard [9]. These breeds have considerably different growth patterns with very small dogs reaching maturity at between 8 and 12 months of age and larger breeds requiring up to 24 months to reach adult body weight [10]. Therefore, unlike the WHO growth standards, it is unlikely that a single growth standard could be created that could be applied to all dogs. Furthermore, whilst previous studies have reported how bodyweight changes during the growth phase for a small number of breeds [10,11,12,13,14], such data are not sufficient for creation of growth curves for all breeds and sizes.

The primary aims of this study were to develop evidence-based growth standards for dogs, using bodyweight data from a large population of healthy pet puppies attending veterinary hospitals in the USA. The secondary aims were to create standards that were applicable to all breeds and sizes of puppy, accounted for sex and neuter status, and could be used to chart healthy growth during early life.

## Materials and methods

### Study design

This was a retrospective observational study using bodyweight and age data from a population of healthy puppies. The study has been reported according to the STrengthening and Reporting of Observational Studies in Epidemiology (STROBE) statement guidelines [15], in accordance with the REporting of studies Conducted using Observational Routinely-collected health Data (RECORD) extension statement (S1 Table) [16].

### Description of the sample population

Banfield Pet Hospitals comprise a network of over 900 primary care veterinary hospitals located mainly in the USA, which have stored patient records electronically since the mid 1990’s. Bodyweight is routinely measured during consultations, whilst breed information is supplied by dog owners but is not verified.

### Data extraction

The proprietary database of electronic patient medical records from Banfield Pet Hospitals was used, henceforth referred to as the 'patient record database'. CS and DW had (read and write) access to a copy of the database (also in Oracle format) containing records up to March 2013, from which client names and their addresses had been removed. Records were available for registered canine patients from this time back to 1994. However, given the growth in client numbers over this time, three quarters of the available data dated from 2003 onwards.

The database was searched for purebred dogs under 3 years of age and additionally either presenting for preventative healthcare reasons or receiving a ‘healthy’ diagnosis (see below for full details). The database queries extracted relevant visit information (patient ID, visit date, visit type, diagnoses received and also age, weight and body condition (if available) of the patient at the time of the visit), together with patient information (patient ID, breed, date of birth and date of neutering [if available]) for all visits by purebred dogs (as evidenced by a species field and the absence of a mixed breed ‘flag’) where the age at visit was under 3 years. Neutering dates had been previously calculated for all dogs neutered at a Banfield Pet Hospital, using details contained in the clinical records, and stored in an additional table. Neutering dates were not available for dogs neutered elsewhere. Given that all variables used for database searching were primary fields, simple search terms were used and no validation of search algorithms were required.

### Eligibility criteria for inclusion into the dataset

Dogs were eligible for inclusion when their data were collected at visits for routine ‘preventative healthcare’, or where the diagnosis was ‘healthy pet’, and where the age (calculated from visit date and date of birth), contemporaneous bodyweight and neuter status of the puppy (calculated from visit date and neuter date, where applicable) could be confirmed for those visits. Data from visits for which any of this information was missing were excluded. Only dogs with at least one bodyweight recorded between the ages of 0.20 years (~10 weeks) and 2.25 years (2 years 3 months) were used for modelling. Dogs that had received a predicted or actual body condition rating other than ‘normal’ or ‘ideal’ (for details, see ‘Generation and Recording of Clinical Data’), or who had received a diagnosis of ‘underweight’, ‘overweight’ or ‘obesity’ at any point up to the age of 3 years were excluded from the dataset. In addition, all individuals needed to have received a predicted body condition rating of ‘normal’ at one or more visits between the ages of 2 and 3 years, since this was taken as an indicator of acceptable body condition having been achieved in young adulthood.

The dog breeds eligible for inclusion are given in Table 1. As a starting point for creating size categories, eligible breeds were initially classified into 5 size classes (toy, small, medium, large and giant) based on a size grouping used in a previously published study [10]. Breeds were assigned to these size classes according to the mean weight across all adult individuals (>2 years old) for that breed in the database. Mixed breed dogs, and those from all other breeds, were excluded.

**Table 1:** Comparison of the pre-existing size classification with the new size categorisation.

Pre-existing size classes^1^			New size categories^2^		
Class	Weight ^(kg)^	Breeds	Category	Weight ^(kg)^	Breeds
Toy	<5	**Chihuahua**, **Yorkshire Terrier**, Maltese, Toy Poodle, **Pomeranian**	I	<6.5	**Chihuahua**, **Yorkshire terrier,** Maltese terrier, Toy Poodle, **Pomeranian**, Miniature Pinscher
Small	5 to <10	Miniature Pinscher, **Shih Tzu**, Pekingese, **Dachshund**, Bichon Frise, Rat Terrier, Jack Russell Terrier, Lhasa Apso, Miniature Schnauzer, Fox Terrier, **Pug**	II	6.5 to <9	**Shih Tzu**, Pekingese, **Dachshund**, Bichon Frise, Rat Terrier, Jack Russell Terrier, Lhasa Apso, Miniature Schnauzer
Medium	10 to <25	**Boston Terrier**, **American Cocker Spaniel**, **Beagle**, Australian Shepherd, Chow Chow, Basset Hound	III	9 to <15	Fox Terrier, **Pug**, **Boston Terrier**, **American Cocker Spaniel**, **Beagle**
Large	25 to 40	Siberian Husky, English Bulldog, **Pit Bull Type**, Boxer, **German Shepherd Dog**, Golden Retriever, **Labrador Retriever**, American Bulldog, Rottweiler	IV	15 to <30	Australian Shepherd Dog, Chow Chow, Basset Hound, Siberian Husky, English Bulldog, **Pit Bull Type** Boxer
Giant	40+	**Great Dane**, **Mastiff**	V	30 to <40	**German Shepherd Dog**, Golden Retriever, **Labrador Retriever**, American Bulldog
			VI	40+	Rottweiler, **Great Dane**, **Mastiff**

^1^ Size classes used in a previously published modelling study [10].

^2^ New size categories, produced after hierarchical modelling.

Bold type denotes breeds for which a breed specific model was created.

At the time of the data extraction, there were 5.5m individual dogs between 10.4 weeks and 2.25 years old in the patient record database, of which 3.8 x10^6^ were purebred. Of these, 3.1 x10^6^ were recorded as being of one of the selected breeds (giving coverage of 57% and 82% of young dogs and purebred young dogs, respectively).

### Generation and recording of clinical data

At the time of initial registration for a puppy, owners supplied signalment data (date of birth, breed [including whether purebred] and sex) which were routinely recorded in the electronic medical records. If a puppy was neutered using a routine surgical procedure (i.e. an elective procedure and not undertaken for health reasons, such as to treat pyometra), this was recorded in the records. Bodyweight was routinely recorded at all visits using 'walk-on' electronic scales. Most Banfield Pet Hospitals use the Weigh South V-2501 large animal veterinary weighing scales (Weigh South Inc, Asheville, North Carolina, USA) after modification by Northwest Scale Systems (Tualatin, OR, USA), with a minority using the VSSI Way™ Platform Scale (VSSI Inc, Carthage, Missouri, USA).

Body condition was examined as part of the eligibility criteria. Historically, body condition was graded using a subjective 3-category body condition assessment (‘thin’, ‘normal’, or ‘heavy’). However, a 5-category body condition score (BCS) scale was introduced in 2010, which was equivalent to the 5-point system used in a previous study [17]. Veterinarians were required to choose one of five categories (e.g. ‘very thin’, ‘thin’, ‘ideal weight’, ‘overweight’, and ‘markedly obese’) after their clinical assessment and with reference to guidance diagrams. However, since the majority of the data extracted used the old 3-category assessment, this information was favoured for analysis. In order to utilise post-2010 data, all 5-category BCS measurements were converted into equivalent 3-category body condition assessments by merging ‘very thin’ and 'thin', and 'overweight' and 'markedly obese'.

Veterinarians assessed body condition at their discretion. If body condition was not assessed, it remained at its default value of 'normal'. To avoid the possibility that such assessments were not an intentional selection from the attending clinician, these values were not used in the analysis, with a predictive model instead being used to predict the most likely BCS. Linear discriminant analysis models were built for 33 common breeds (based upon number of dogs under 2 years old in the database) to predict BCS in adult dogs from actual weight, recommended weight and demographic factors (sex, neuter status, age and breed). Predictions where no category had a certainty of over 60% were replaced with ‘unknown’. These models were tested on unseen validation datasets and were found to have acceptable accuracy (70–75%, depending on breed). However, such models were not built for puppy visits because of the added complexity of predicting body condition while the individual was still growing. Therefore, for visits under 2 years old where the body condition was left at the software’s default value, the BCS was replaced by ‘unknown’. One final validation check was to review the diagnosis field in the electronic medical record, where it is possible for veterinarians to record an actual diagnosis of ‘obesity’, ‘overweight’ or ‘underweight’. Where this was the case, the BCS record was reviewed and, if necessary, amended to reflect the diagnosis.

### Data handling and statistical analysis

#### Sample size

A formal sample size calculation was not performed. Instead, the aim was to include as many dogs as possible that met the eligibility criteria. The dataset available was at least as large as those used in other studies to create growth charts [18], and the larger datasets used were comparable (in terms of the number of data points) to those used by the WHO Multicentre Growth Reference Study Group to construct the WHO Child Growth Standards [19].

#### Data cleaning

The datasets were cleaned by first excluding extreme upper outliers (i.e. more than 3 times the median weight for individuals over 1yr old). Bodyweight was then split into 40 equal-size age groups and plotted as box-and-whisker plots [20]. Loess (smoothed) regression lines, with a smoothing span of 0.8, were fitted through the upper and lower outlier limits of each bin (defined as 150% of the upper and lower whiskers), and points outside these lines were excluded. Additional data cleaning was implemented for dogs with repeat visit data (‘within dog cleaning’), where it was possible to check plausibility of recorded bodyweights against previous and subsequent measurements for the same animal. To do this, bodyweights were converted to z-scores (i.e. converted to a standard deviation scale, oriented at the median) using the appropriate initial growth curve model, and then the distance of each point from the mean of the remaining points for that dog was calculated as a multiple of the standard deviation of those remaining points. All data points where this multiple was greater than 3 were assumed to be outliers and were excluded.

Small numbers of observations were also removed from the datasets for these final models on the basis that the bodyweights had apparently been rolled over from previous visits (which is sometimes done in Banfield hospitals if the scale is unavailable when the pet is checked in), the dog became pregnant, or there was some doubt over the recorded sex (for example, a female dog booked in for a castration).

#### Creation of growth centile curves

Growth centile curves were constructed using Generalised Additive Models for Location, Shape and Scale (GAMLSS) [21], the same model class that the WHO Multicentre Growth Reference Study Group used to construct the WHO Child Growth Standards [2]. GAMLSS is a semi-parametric modelling technique, whereby aspects of the underlying distribution (central tendency, spread, skewness and kurtosis) are estimated as smooth functions of the predictor variable(s). All analysis was performed with R3.1.1 [22], using the R package gamlss [21]. Creation of the growth centile curves was a multi-stage process and further criteria were applied specifically to certain stages, as outlined below. The variables used in the models were age and bodyweight. Age was raised to the power of 0.1 before modelling as this best improved the fit of the resulting models out of a range of powers tried between 0.001 and 2. Separate models were built for diverse combinations of demographic factors, including breed, sex and neuter status / neuter age, at different stages in the project, in order to investigate the effects of neutering and breed size (see below).

#### Choice of model

Two particular GAMLSS models were considered: the BCCG (Box-Cox Cole-Green) model, which models central tendency, spread and skewness, and the more complex BCPE (Box-Cox Power Exponential) model, which additionally models kurtosis and has the BCCG model as a special case (when kurtosis is absent).

#### Smoothing techniques

The functions for location, scale, skewness and kurtosis were smoothed with penalised beta splines, using the local Generalised Akaike Information Criterion [23] to estimate the most appropriate value for the degrees of freedom. This was done using the pb() function in the gamlss R package [21]. Smoothing parameters were chosen by assessing model fit and suitability across a range of values, with a focus on those lying between the Akaike Information Criterion (AIC) and the Schwarz Bayes Criterion (SBC), as recommended by Rigby & Stasinopoulos [24].

#### Modelling strategy

The BCPE model was fitted, initially using the SBC as smoothing criterion for all parameters (i.e. central tendency, spread, skewness and kurtosis). Model fit was examined using techniques described previously [25], including residual plots, normal score plots and worm plots. To achieve an acceptable fit, models were successively refitted adjusting the smoothing spline degrees of freedom. If the kurtosis function was largely flat the model was compared to the simpler BCCG model. Avoiding over-fitting was prioritised over improving goodness of fit [25].

#### Plotting of growth centile curves

The models were displayed graphically as centile curves covering the age range 12 weeks to 2 years, and showing centiles 0.4%, 2%, 9%, 25%, 50%, 75%, 91%, 98% and 99.6%. These centiles are the same as those used in the UK-WHO growth charts [26], and they are also equally spaced on the z-score (standard deviation) scale, which is advantageous from an arithmetic point of view.

Models were initially fitted for purebred dogs of 8 breeds (American Cocker Spaniel, Beagle, Chihuahua, German Shepherd Dog, Labrador Retriever, Pomeranian, Shih Tzu and Yorkshire Terrier), and also for the 'Pit Bull type' given that this was common, in terms of numbers of dogs within the database. Additional models for intact dogs (only) were added later for a further 5 breeds (Boston Terrier, Dachshund, Great Dane, Mastiff and Pug). Separate charts were constructed for males and females, and for intact dogs and 4 neutering age groups, which were chosen to be of approximately equal size across all breeds (0 to <22 wks, 22 to <26 wks, 26 to <37 wks and >37 wks). These charts were then used to address the effects of neutering, breed and size (using adult body weight) on growth.

#### Methods used to address effects of neutering during chart development

The initial models for the original 9 breeds were used to assess the effects of neutering on bodyweight in two ways. The first involved a visual comparison of centiles for intact and neutered dogs, whilst the second involved modelling changes in centiles after neutering. For the visual comparison approach, for each breed × sex combination, the bodyweights for all neuter-age groups were converted to z-scores based on the model for intact dogs. These were plotted against age and assessed visually, the x-axis indicating the intact group median, and a positive/negative slope indicating an increasing/decreasing centile trend in the neuter group.

For the modelling approach, a subset of the data was selected, comprising dogs where the neutering age was known (henceforth referred to as ‘baseline’) and at least one post-neutering bodyweight measurement had been recorded. Dogs were excluded when their baseline z-score was greater than 3, the difference between the baseline centile and the average centile of the post-neutering visits divided by the standard deviation of the centiles was greater than 5, or a post-neutering visit centile in the first 4 weeks differed from the baseline centile by >40% (absolute). GAMLSS was then used to model post-neutering changes in z-score in terms of the number of weeks from the neutering event. A t-family distribution was used for the residual distribution, and a Box-Cox transformation was applied to the dependent variable where it improved fit. The starting model for the stepwise GAMLSS allowed the models for location and spread to be a smooth function of ‘weeks since neutering’ whilst the degrees of freedom was modelled as a numerical constant term. The most complex model allowed to the stepwise GAMLSS included all of the location, spread and degrees of freedom as smooth functions of 'weeks since neutering', 'neuter age' and 'model z-score at neutering', with interactions up to second order. The least complex model allowed had all three terms as numerical constants. The smooth functions were fitted using p-splines (penalised beta splines), with local maximum likelihood used to choose the degrees of freedom for the smoothing. Interaction terms were fitted as varying coefficient models. The possibility that any of the smooth functions in the finished model could be simplified to linear functions was then checked manually. The model was used to calculate median predicted changes from baseline together with the associated interquartile ranges for a dog on the 50^th^ centile at the time of neutering, at successive 1 week intervals from neutering to 2 years old. The four neuter-age ranges were represented by neutering ages of 22 wks, 26 wks, 37 wks and 52 wks. Plots of these data (by breed, sex and neuter age) were then examined visually and the interquartile ratio (interquartile range divided by the median, a non-parametric equivalent to the coefficient of variation) was calculated at each age. This enabled the size of the predicted spread to be summarised relative to the size of the predicted change (at the level of breed, sex and neutering age).

#### Comparison of growth curves modelled on breed with growth curves modelled on adult size

Our overall aim for these comparisons was to determine whether growth could successfully be modelled on size (using adult body weight) irrespective of breed, or whether it was essential to build breed-specific charts. A subset comprising data from intact dogs from 33 common breeds was extracted. Growth centile curves were then constructed for groups of breeds in the same adult size category (Table 1), initially defined to be the pre-existing size classes [10]. These were then compared with the breed-specific models, and the size categories subsequently adjusted as necessary. Size category models were assessed against the breed-specific models for breeds in that size category, and it was assumed that the breed models were more accurate. Predicted weight trajectories from the two models were compared graphically, for theoretical dogs situated on the standard centiles of the breed-specific model at 3 or 6 months of age (the ‘reference age’). An additional comparison was made with the size category model refitted on data excluding the breed under examination, to check for undue influence of the breed on the size category. The level of agreement was designated as acceptable if none of the trajectories crossed neighbouring centile lines.

Initial adjustments to the size categories were made when anomalies in the residuals or indications of poor fit in the size category models were identified. Further refinements were then made on the basis of hierarchical cluster analyses (using a Euclidean distance metric with average linkage, and undertaken separately for males and females) of growth trajectories constructed from loess smoothed median weights at 29 age points (0.25 yrs, 0.30 yrs, 0.35 yrs, …, 1.5 yrs, 1.6 yrs, 1.8 yrs, 2 yrs). This analysis was repeated for 2 different sets of data: set 1 comprised 33 breeds in the main dataset (cleaned as previously described) whilst set 2 comprised an extended set of 73 breeds for which there were visits recorded for over 5000 purebred individuals under 2 years old. The data in set 2 were cleaned as for set 1 with respect to visit reason or diagnosis, but were not cleaned with respect to body condition score (due to the BCS model not being available for all the breeds in this extended set). Therefore, set 1 was a smaller dataset with fewer breeds, but excluded dogs whose body condition was unlikely to be ideal. In contrast, set 2 was larger, with more breed coverage, but the data may have included overweight and underweight individuals. When constructing the new size categories, set 1 was considered to have priority over set 2 in the case of disagreement. The agreement between the size category models and breed-specific models was then rechecked as above.

## Results

### Sample population

**Fig 1:**
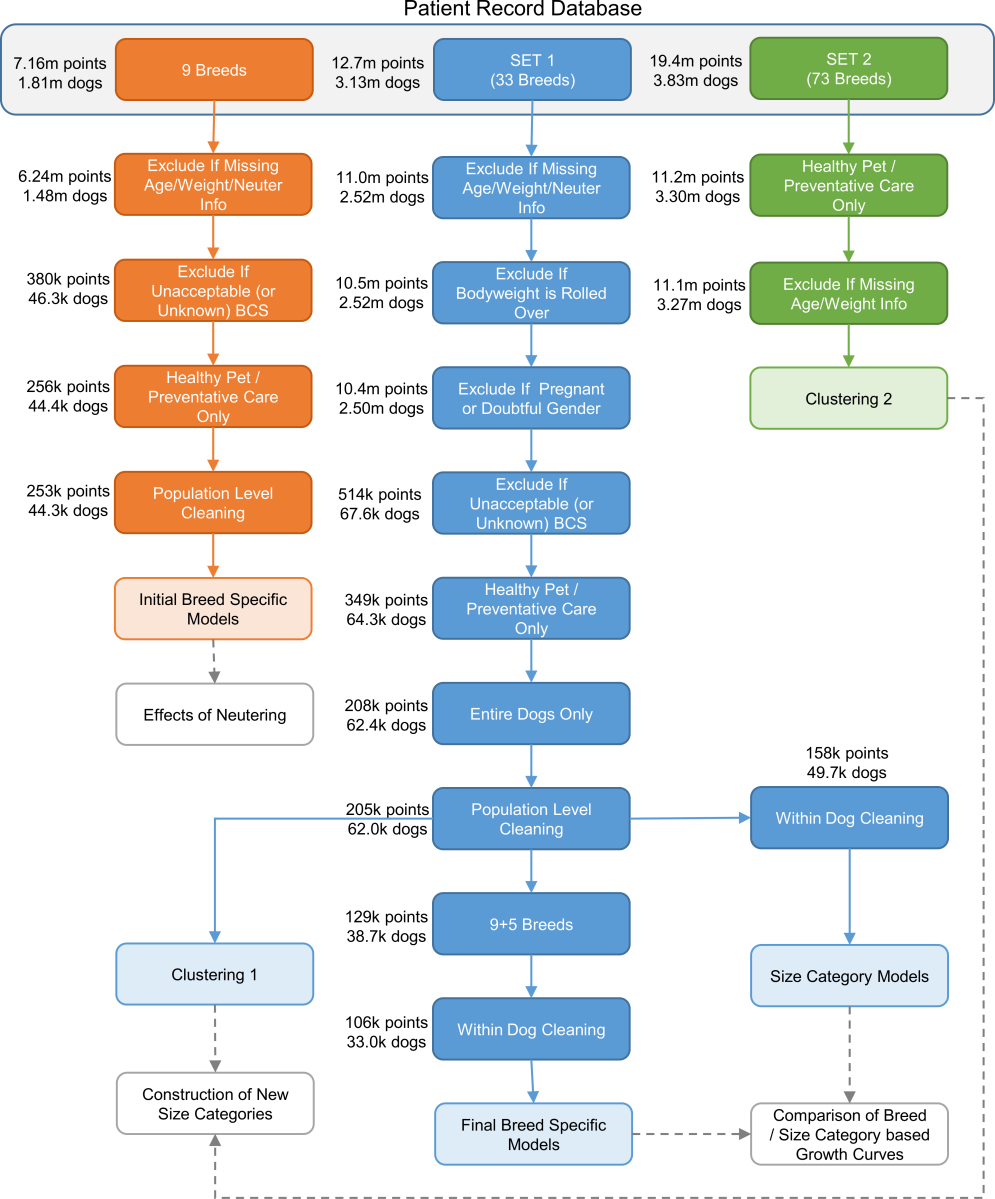
Data cleaning process. Flow diagram illustrating the data cleaning process for datasets used in the creation of growth curves and steps involved in constructing the different datasets (light blue boxes) from the original anonymised patient record database.

There were 5 main datasets used in the analyses: the dataset for the initial breed-specific models, the dataset for the final breed-specific models, the dataset for the final size category curves, and the datasets for the two clustering analyses. The final dataset sizes are summarised in Table 2, whilst Fig 1 illustrates the steps in constructing these datasets and shows the number of observations and dogs remaining in the dataset at each stage.

**Table 2:** Final sizes of the 5 main datasets used and their associated sub-datasets.

Dataset	Total Dataset Size		Sub-Datasets			
	Data points^1^	Dogs^1^	No.	Type	Data points^2^	Dogs^2^
Initial Breed Specific Models	2.53 x10^5^	4.43 x10^4^	90	9 Breeds × 2 Sexes × 5 Neuter groups	5.40 x10^3^ (575 to 1.87 x10^4^)	1.45 x10^3^ (79 to 5.57 x10^3^)
Final Breed Specific Models	1.06 x10^5^	3.3 x10^4^	30	15 Breeds × 2 Sexes	3.57 x10^3^ (241 to 1.13 x10^4^)	1.16 x10^3^ (78 to 3.52 x10^3^)
New Size Category Models	1.58 x10^5^	4.97 x10^4^	12	6 Breed sizes × 2 Sexes	16.6 x10^3^ (8.75 x10^3^ to 2.13 x10^4^)	5.25 x10^3^ (3.00 x10^3^ to 6.49 x10^3^)
SET 1 Clustering	2.05 x10^5^	6.2 x10^4^		N/A	N/A	N/A
SET 2 Clustering	1.11 x10^7^	3.27 x10^6^	N/A	N/A	N/A	N/A

^1^ Total number of datapoints and dogs within the respective dataset.

^2^ Median (range) number of datapoints and dogs within each of the data subsets used in the modelling.

### Construction of breed specific growth curves

Growth centile curves were successfully constructed for each of the 100 breed-specific models examined (i.e. 5 neuter categories × 2 sexes × 9 original breeds, plus 1 neuter category [intact] × 2 sexes × 5 further breeds). All the curves constructed were based on the BCPE model, and the vast majority (82 models) used the SBC as the smoothing criterion for all four parameters.

### Comparison of growth curves for neutered and intact dogs

#### Visual comparison

**Fig 2:**
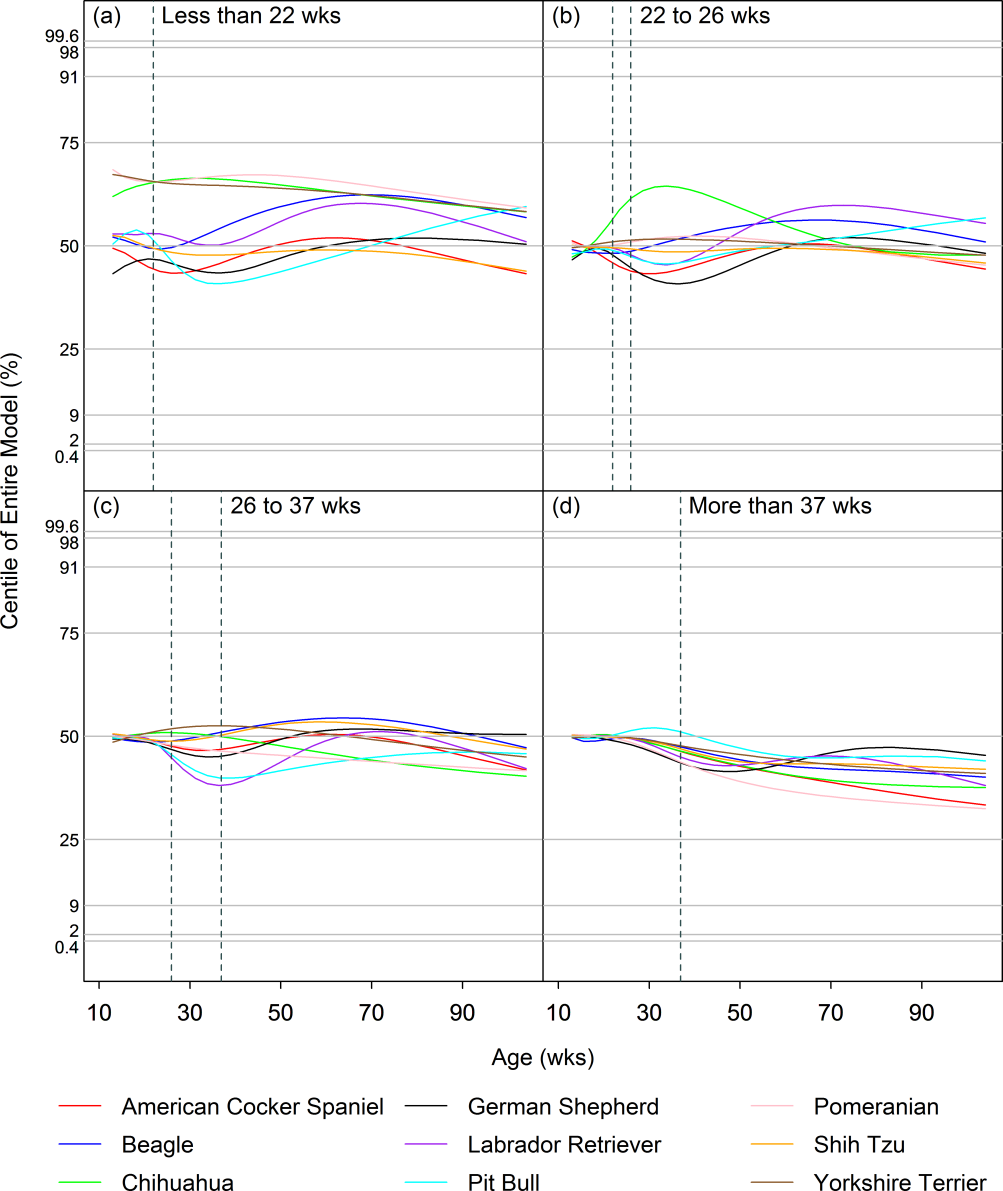
Effect of neutering on growth in male dogs. Median (50^th^ centile) curves for males of each of the 9 breeds for each neuter age group (a: <22wks; b: 22-26wks; c: 26-37wks; d: >37wks), after conversion to z-scores using the appropriate entire group model. The vertical dashed lines represent the timing of neutering in each of 4 groups. The horizontal dashed lines indicate the standard centiles (0.4%, 2%, 9%, 25%, 50%, 75%, 91%, 98% and 99.6%). A horizontal plotted curve represents exact correspondence between the neuter group centile and the intact group centile whilst, at that any given age, a positive or negative slope indicates an increased or decreased weight in the neuter group centile compared with the intact group centile, respectively.

**Fig 3:**
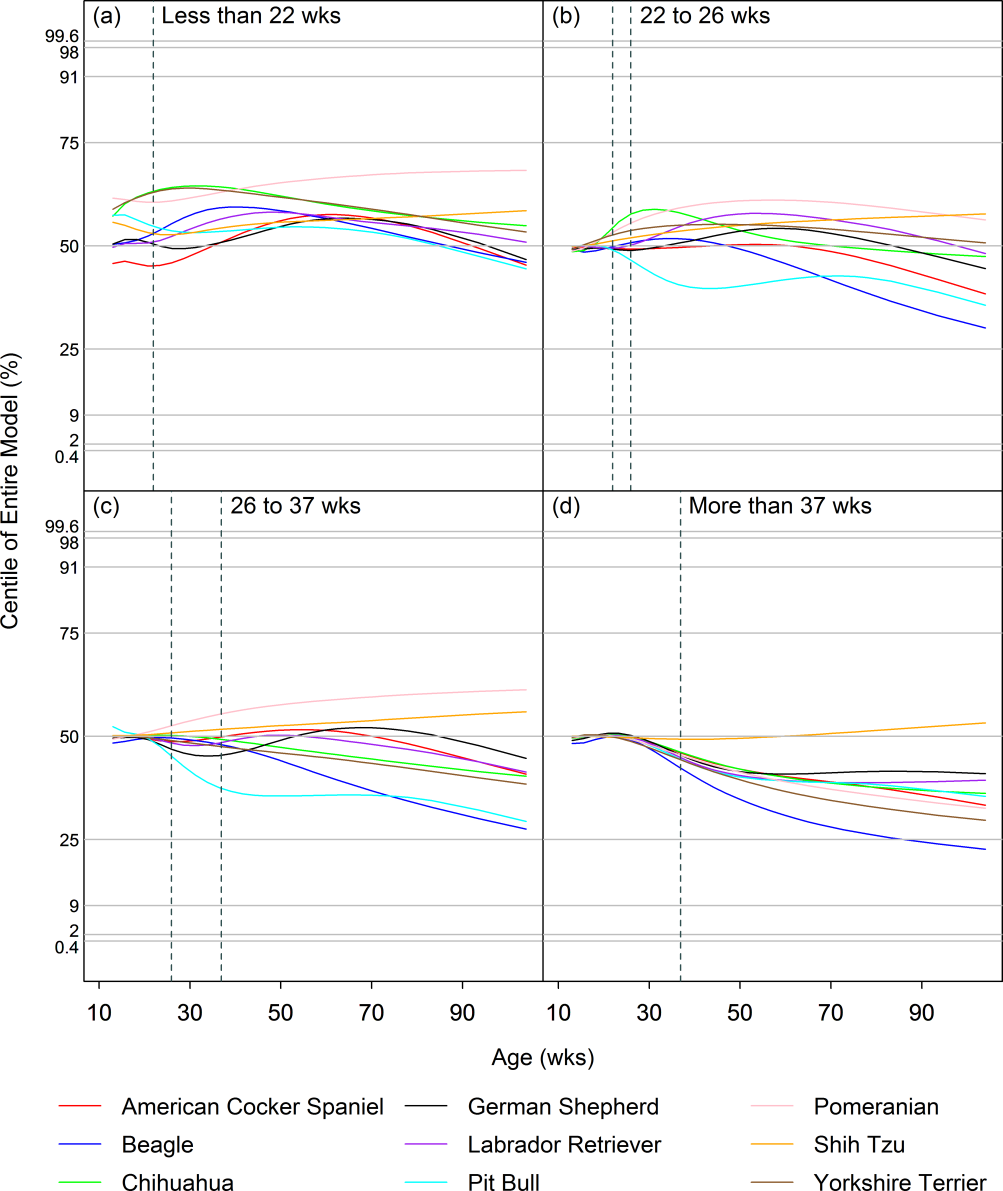
Effect of neutering on growth in female dogs. Paths of the median (50%) centile curves for females of each of the 9 breeds for each neuter age group (a: <22wks; b: 22-26wks; c: 26-37wks; d: >37wks), after conversion to z-scores using the appropriate entire group model. The vertical dashed lines represent the timing of neutering in each of 4 groups. The horizontal dashed lines indicate the standard centiles (0.4%, 2%, 9%, 25%, 50%, 75%, 91%, 98% and 99.6%). A horizontal plotted curve represents exact correspondence between the neuter group centile and the intact group centile whilst, at that any given age, a positive or negative slope indicates an increased or decreased weight in the neuter group centile compared with the intact group centile, respectively.

Figs 2 and 3 illustrate the median (50^th^ centile) curves for each of the 9 breeds for each neuter age group, for males and females respectively, after conversion to z-scores using the appropriate intact group model. As illustrated, changes in the rate of growth associated with neutering created slight differences in the centile curves for most breeds, but they were too small to cause centile lines to cross over neighbouring lines. Indeed, centile crossing was seen in only 4/64 instances and never crossed more than 1 neighbouring curve.

Neutering between 22wks and 26wks, and between 26wks and 37wks of age, was associated with various patterns of change, depending on breed, but a short-lived upwards shift (of <1 centile) with subsequent return to the original trajectory was most commonly seen (Figs 2 and 3). The effects of neutering before 22wks were similar, except that the upward shifts in growth trajectory were more pronounced. However, small differences (e.g. up to half a centile channel width) in growth trajectories for neutered and intact dogs were sometimes evident even by 12wks of age (the lower age limit of the growth curves), and especially noticeable in the smaller breeds. This was not the case for the other neuter age groups and suggests that dogs that are neutered early may be a different, slightly heavier population, complicating the interpretation for this group. Finally, late neutering (after 37wks of age) led to small (<1 centile) decreases in trajectory of centile curves for almost all of the breed × sex combinations.

#### Modelling differences in centiles post-neutering

When differences in centiles were modelled post-neutering, the general trends in growth trajectory observed were similar to those identified by visual assessment. However, the downwards deviation in growth trajectory was less evident for neutering after 37 weeks of age. In addition, for all the examined breed × sex combinations, the interquartile ratio was large (Table 3), meaning that the spread of the population around the predicted change in z-score was wide in comparison to the magnitude of the predicted change. Examination of individual plots confirmed this observation and also that the interquartile ranges included the lines of zero change. This suggests that dog-to-dog variability was large compared with the magnitude of the post-neutering change in median weight predicted by the models. For most breeds, the earlier the neutering was performed, the more variable its effect.

**Table 3:** Interquartile ratio of predicted post-neutering changes in z-score for a dog on the 50^th^ centile at neutering.

Breed	Sex	Neuter Age Group			
		up to 22wks	22wks to 26wks	26wks to 37wks	37wks onwards
American Cocker Spaniel	M	16 (10, 30)	14.5 (9.81, 29.8)	10.9 (8.23, 19.4)	7.67 (6.54, 10.2)
	F	8.5 (7, 14)	8.6 (6.9, 14)	9.1 (6.9, 15)	7.3 (6, 12)
Beagle	M	4.7 (4.1, 5.6)	5.4 (4.7, 6.6)	7.9 (6.5, 12)	16 (11, 39)
	F	7.5 (4.1, 19)	5.8 (4.1, 8)	5.7 (4.2, 8.8)	5.2 (4, 17)
Chihuahua	M	6 (4.6, 18)	6.3 (4.5, 18)	7.5 (4.8, 23)	9.9 (7.9, 29)
	F	13 (8.1, 20)	13 (8.2, 20)	13 (8.7, 20)	13 (6.6, 22)
German Shepherd Dog	M	40 (28, 81)	40 (27, 79)	43 (29, 87)	49 (32, 98)
	F	24 (16, 51)	25 (16, 50)	24 (16, 48)	28 (18, 55)
Labrador Retriever	M	16 (11, 23)	16 (10, 22)	12 (8.6, 17)	8.8 (6.8, 14)
	F	10 (6.2, 33)	9.2 (5.8, 28)	6.9 (5, 15)	5.1 (4.1, 7.5)
Pit Bull Type	M	24 (15, 47)	24 (15, 45)	22 (16, 44)	22 (15, 44)
	F	7.4 (5.9, 10)	4.6 (4.2, 5)	2.5 (2.4, 2.5)	2.5 (2.4, 2.6)
Pomeranian	M	21 (14, 45)	22 (15, 47)	26 (17, 56)	35 (22, 71)
	F	32 (16, 63)	34 (15, 67)	33 (13, 78)	21 (11, 52)
Shih Tzu	M	18 (7.6, 45)	12 (7.2, 29)	8.5 (7.3, 20)	3.9 (3.1, 11)
	F	9.3 (8.1, 12)	9 (7.4, 12)	8.2 (5.8, 12)	7.6 (4.2, 11)
Yorkshire Terrier	M	8.3 (5.4, 18)	8.6 (5.8, 19)	9.9 (7, 23)	13 (8.6, 29)
	F	7.6 (5.3, 21)	8 (5.4, 23)	8.9 (5.8, 27)	12 (6.9, 35)

All numerical data reported are interquartile ratios of predicted post-neutering change in z-score. The first number indicates the median interquartile ratio, with the interquartile range being displayed in brackets. Four columns of data are reported, each corresponding to a different age of neutering to 2 years of age. For each neuter-age group, separate results are reported for male (M) and female (F) dogs in different breeds. The interquartile ratio is calculated by dividing the interquartile range by the median. The larger the number the greater the degree of inter-individual variation relative to the median population trend. For example, if the median z-score was 0.1 and the interquartile range varied from -0.1 to 0.2, the interquartile ratio would be 3. In contrast, a median z-score of 0.05 and interquartile range between -0.3 and 0.4 would mean an interquartile ratio of 14.

### Comparison of growth curves modelled on breed and adult size

All the size-category models (both those using the pre-existing size classes and those based on the later adjusted size categories, henceforth referred to as 'new size categories') were based on the BCPE model and used the SBC as the smoothing criterion for all four parameters. Given the variability in response to neutering, and possible variability in rates of neutering amongst breed, all comparisons were made solely with data from intact animals.

When using the pre-existing size classes, several issues were noted in the modelling analysis as indicated either by anomalies in the residuals or poor model fit within the size category. Firstly, model residuals for the medium size class were strongly bimodal, with the subpopulations corresponding to breeds <15kg and breeds >15kg, suggesting that this size category needed splitting. Secondly, the Rottweiler breed, which was the heaviest of the large breeds examined, fitted poorly and was excessively influential, suggesting that the upper boundary needed shifting down. Finally, the Fox Terrier and Pug breeds, which were the largest of the small breeds, were excessively influential within their category, again suggesting that the upper boundary be shifted down.

**Fig 4:**
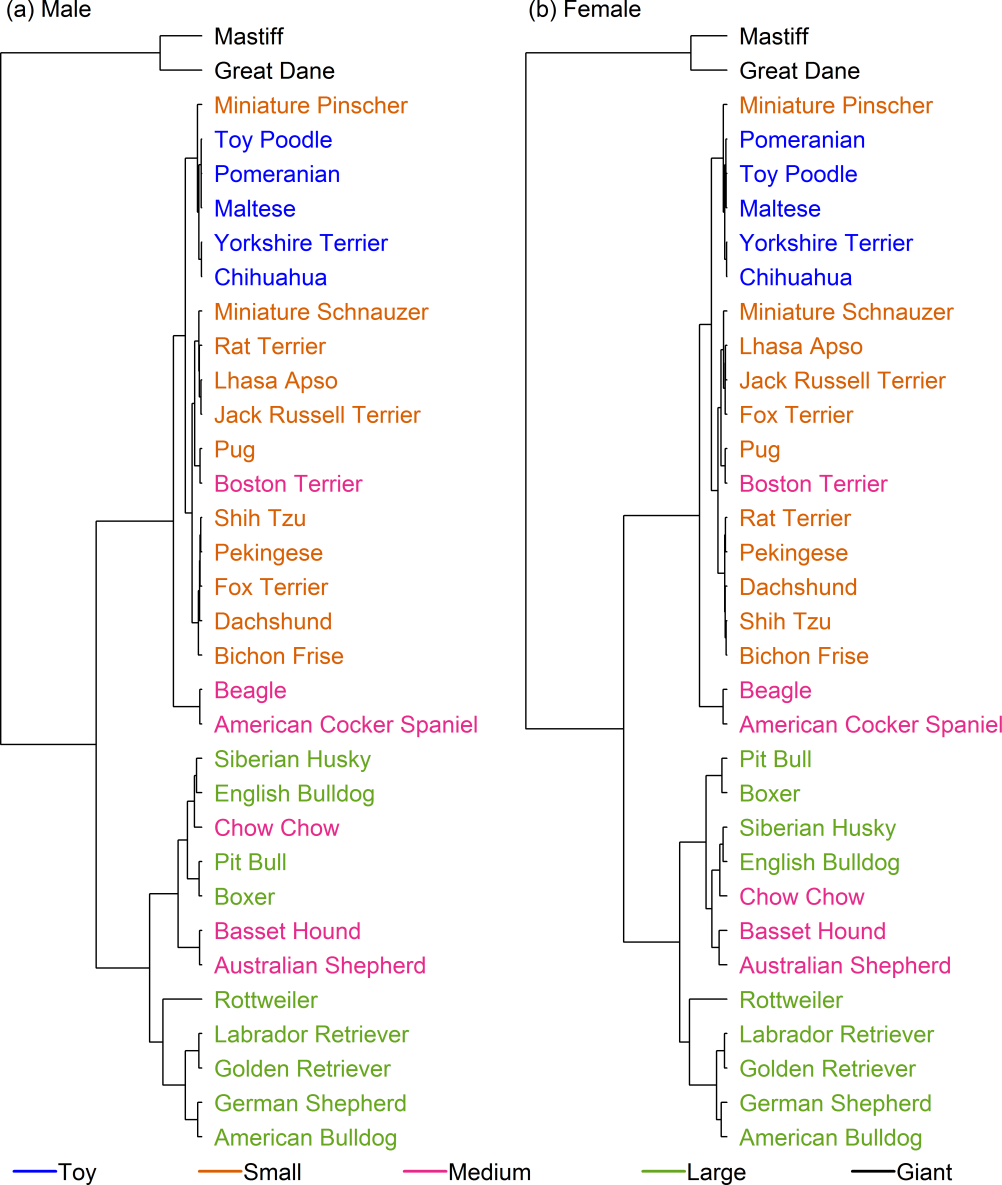
Hierarchical clustering of dog breeds in set 1. Dendrogram illustrating the hierarchical clustering conducted on the median growth trajectory of 33 breeds (set 1) for male (a) and female (b) dogs. The earlier the clade (branch) occurs, the more dissimilar the breeds are from one another. Most notably, two giant breed dogs (Great Dane and Mastiff) branch early, suggesting a markedly different growth pattern from other dogs, whilst the Rottweiler is the only breed in a single-breed cluster, having branched early from other 'large' breeds. The breeds are colour-coded according to the pre-existing size categories (as shown in Table 1, [10]).

Further refinements in breed categorisation were made using cluster analysis employing two sets of data. Fig 4 shows the dendrograms for the cluster analysis on set 1 (the smaller but cleaner dataset), coloured by pre-existing size class. The cluster analyses for male and female were broadly similar. In several places, it was noted that growth patterns of breeds were not consistent within the allocated size categories. For example, the 'Giant' breeds (Great Dane and Mastiff) were the first breeds to branch, indicating a markedly different growth pattern, whilst the 'lightest' breeds within the large size class (e.g. Boxer, English Bulldog, Siberian Husky, and the Pit Bull type) clustered more closely with heavier breeds in the medium size class (e.g. Australian Shepherd, Bassett Hound, and Chow Chow) than with other large breeds. Further, Rottweiler branched early from other breeds within the large size class, indicating a different growth pattern, whilst the growth patterns of the ‘small’ Pug more closely matched those of the ‘medium’ Boston Terrier than those of the other small breeds. Finally, the growth trajectory of the Miniature Pinscher (the lightest breed within the small size class) clustered more closely with breeds in the toy size class.

**Fig 5:**
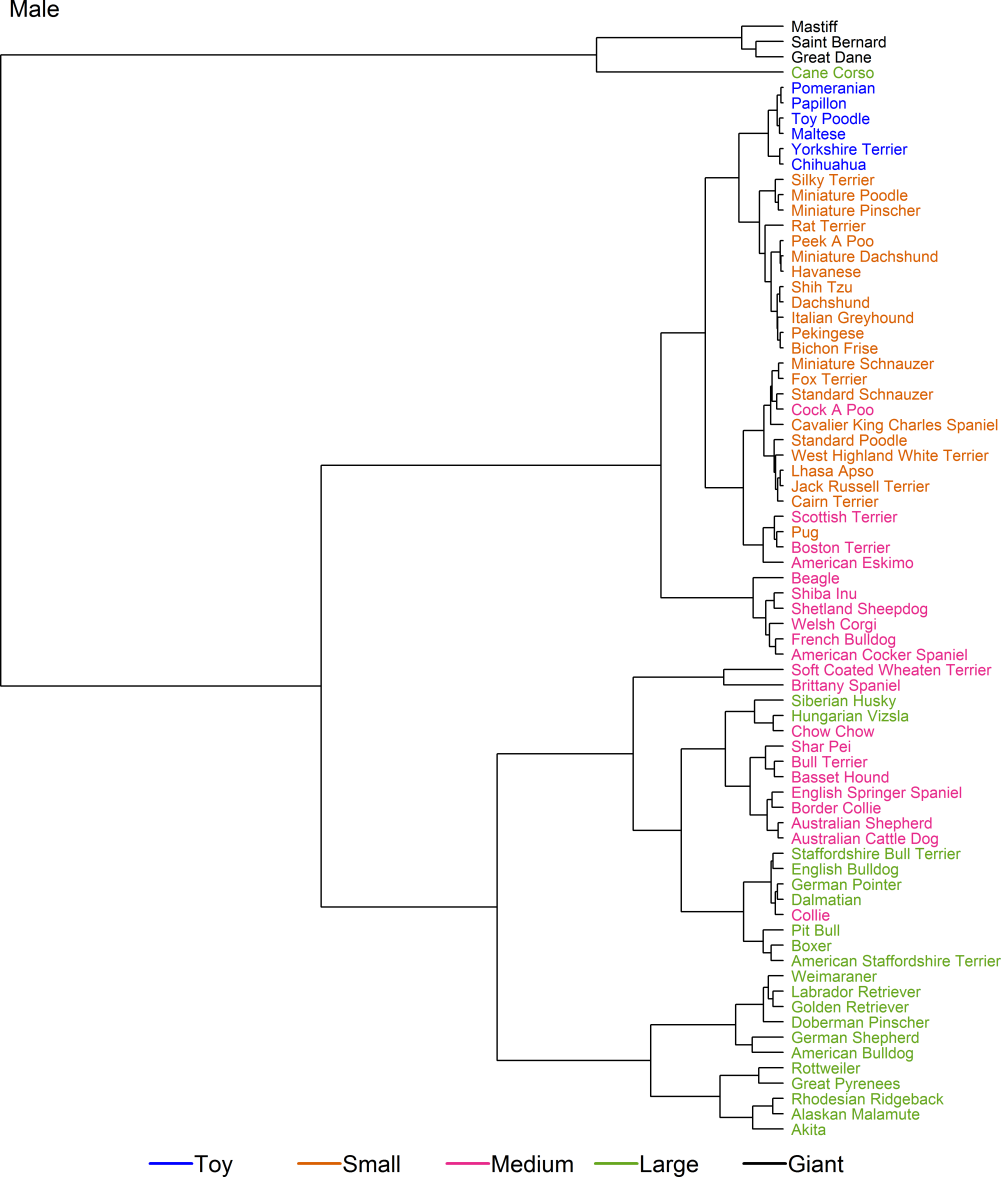
Hierarchical clustering of male dogs from various breeds in set 2. Dendrogram illustrating hierarchical clustering conducted on the median growth trajectory of male dogs of 73 breeds (set 2). The earlier the clade (branch) occurs, the more dissimilar the breeds are from one another. A range of different sub-clusters are highlighted but, most notably and similar to the results of the analysis on set 1 (Fig 4), the growth trajectories for three breeds in the giant-breed category (Saint Bernard, Mastiff and Great Dane) divide early from the other breeds, and cluster with the Cane Corso breed. The breeds are colour-coded according to the pre-existing size categories (as shown in Table 1, [10]).

**Fig 6:**
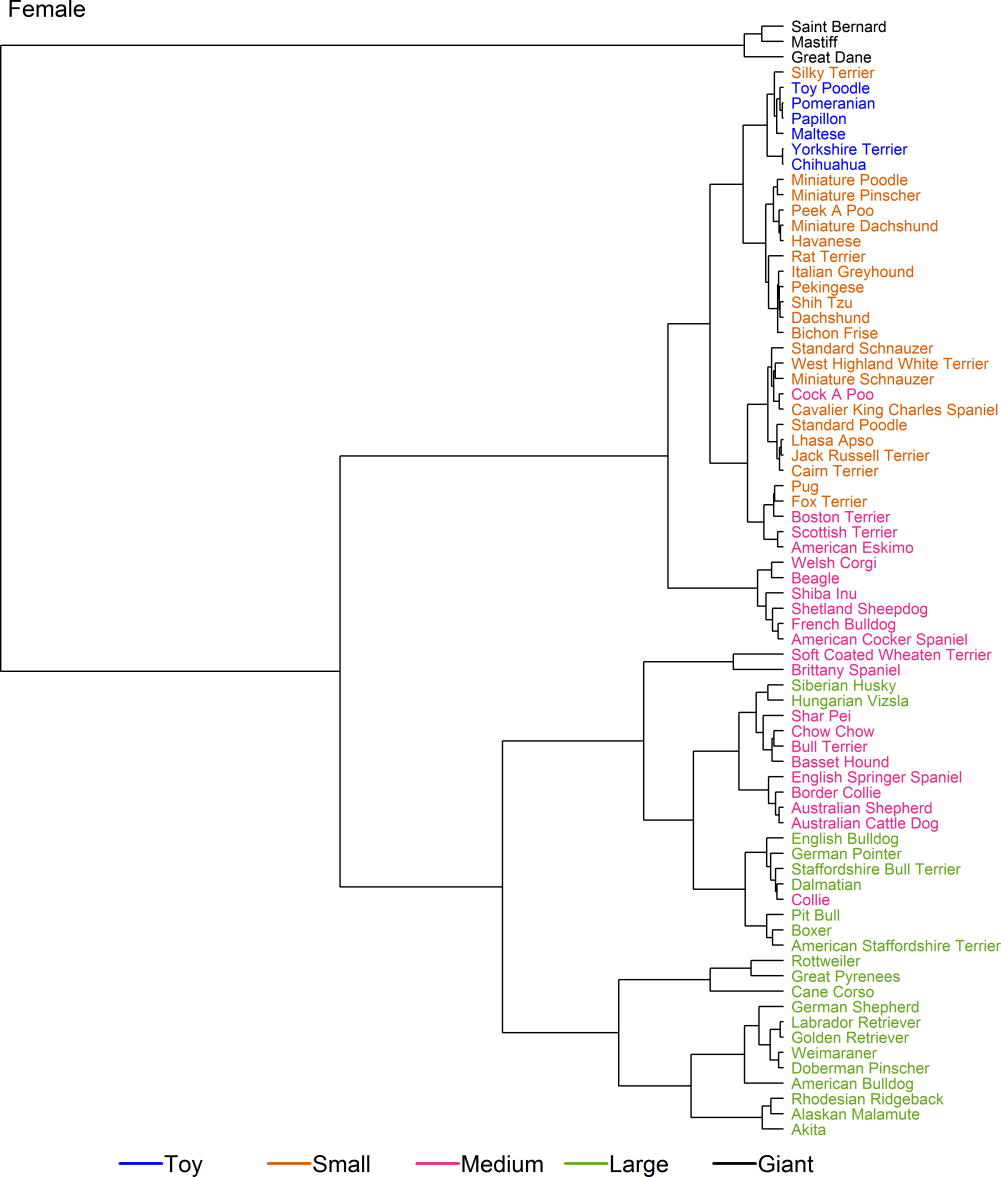
Hierarchical clustering of female dogs from various breeds in set 2. Dendrogram illustrating hierarchical clustering conducted on the median growth trajectory of female dogs of 73 breeds (set 2). A range of different sub-clusters are highlighted, with a broadly similar pattern to that seen in male dogs from the same dataset (Fig 4). Growth trajectories for three giant-breed category dogs (Saint Bernard, Mastiff and Great Dane) again separate earliest from other dogs but, unlike male dogs, did not cluster with the Cane Corso breed which, instead, clustered more closely with Rottweiler and Great Pyrenees. The breeds are colour-coded according to the pre-existing size categories (as shown in Table 1, [10]).

Figs 5 and 6 show dendrograms on set 2 (the larger but less clean dataset) for male and female dogs, respectively. Once again, male and female dendrograms were similar. As for set 1, the growth trajectories for breeds in the giant size class (this time Saint Bernard, Mastiff and Great Dane) branched early. Secondly, Pug, Boston Terrier, Fox Terrier, Scottish Terrier and American Eskimo clustered together with an expected adult weight between 9 kg and 11 kg, these breeds straddled the boundary between the original small and medium size classes and formed a 'sub-cluster' that included Cairn Terrier, Cavalier King Charles Spaniel, the 'Cockapoo' breed type, Jack Russell Terrier, Lhasa Apso, Miniature Schnauzer, Standard Poodle, Standard Schnauzer, and West Highland White Terrier. All the other breeds within the original small and toy size class made up a second sub-cluster, suggesting that upwards adjustments of the upper limits for both the small and toy size classes were required. A third sub-cluster of this part of the dendogram comprised breeds from the middle of the original medium size class (e.g. American Cocker Spaniel, Beagle, French Bulldog, Shetland Sheepdog, Shiba Inu and Welsh Corgi), whilst a fourth sub-cluster combined the heavier breeds of the original medium size class (from Soft-Coated Wheaten Terrier to Chow Chow) and the lighter breeds from the large size class cluster (from English Bulldog to Boxer). The remaining breeds from the large size class (from Weimaraner to Rottweiler) formed a final cluster.

### New size categorisation

Taken together, the initial modelling and subsequent cluster analysis suggested that a reorganisation of categories was required, with a splitting of the original medium size class to form an additional category and some alterations to the boundaries of the remaining categories. This ensured that none of the groupings had a bimodal distribution or contained breeds which were excessively influential on model fit. To distinguish between this new size categorisation and the pre-existing size classes, the new categories were named using Roman numerals (i.e. I, II, III, IV, V and VI, from smallest to largest), as shown in Table 1. For these new size categories, the breeds used in the modelling accounted for between 39% and 78% of visits by dogs under 2 years of age recorded by Banfield Pet Hospitals since 2002, depending on breed, and between 58% and 91% of visits by purebred dogs.

### Assessment of new size-specific vs. breed-specific models

For most breeds, the new size category models and breed-specific models gave more consistent predictions for a reference age of 6 months than for a reference age of 3 months. The most common breeds in size category VI (e.g. Great Dane, Mastiff and Rottweiler) had widely varying growth trajectories and, consequently, it was not possible to create a growth curve model for this category, even at the later reference age. As a result, all subsequent work was conducted on the other size categories (I-V).

**Fig 7:**
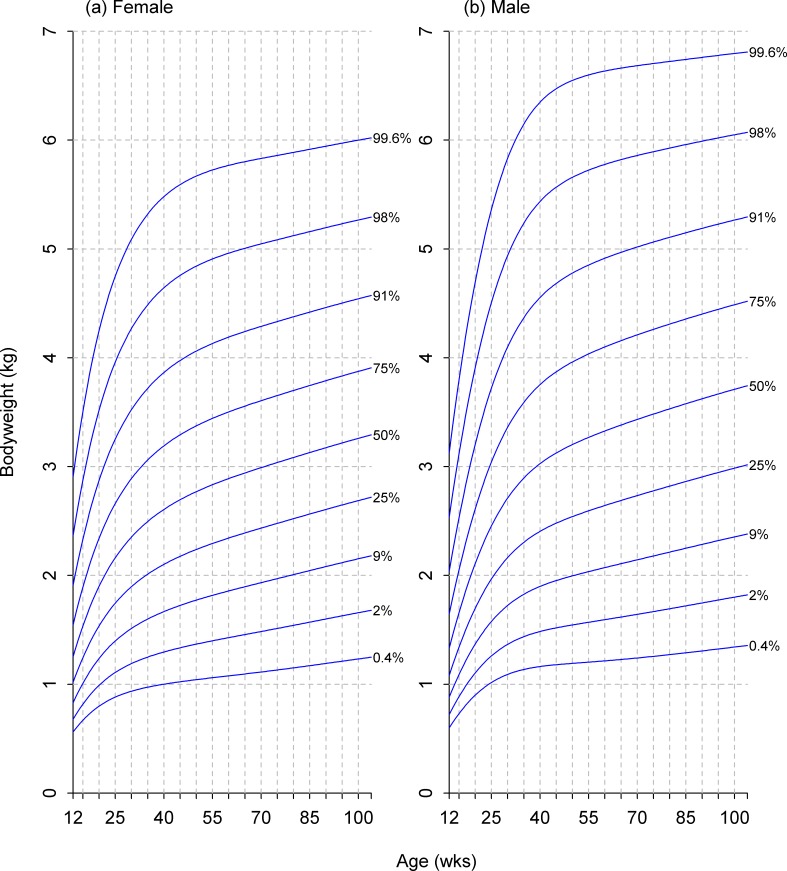
Growth standard chart for size category I. Left and right panels show the final curves for males and females, respectively, for dogs with a predicted adult bodyweight of <6.5kg. The x-axis depicts age in weeks, whereas the y-axis depicts bodyweight in kilograms.

**Fig 8:**
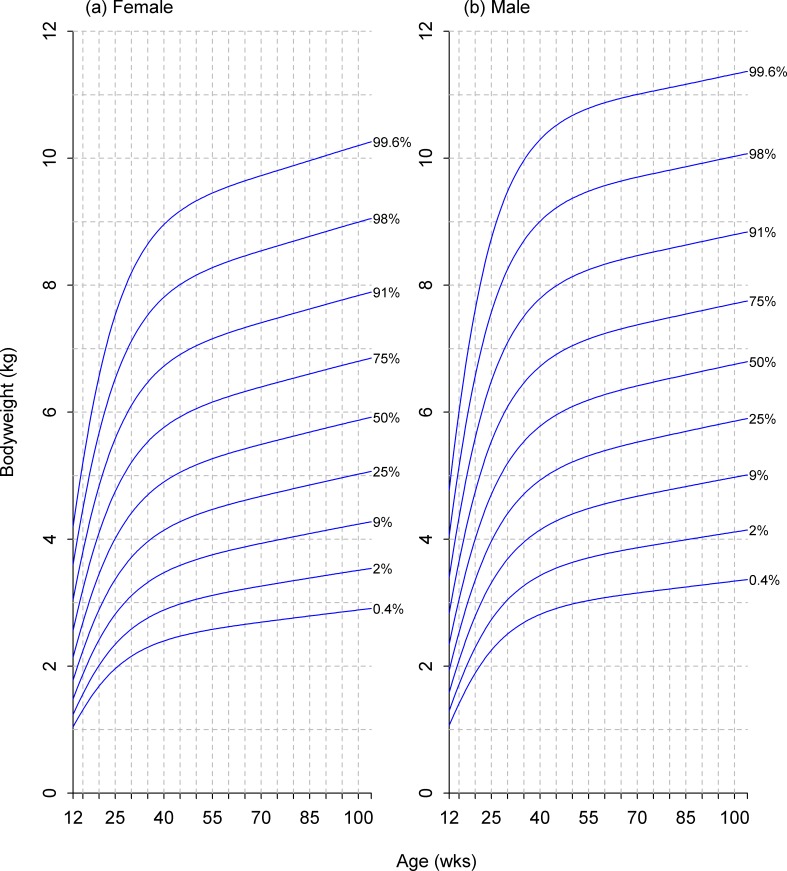
Growth standard chart for size category II. Left and right panels show the final curves for males and females, respectively, for dogs with a predicted adult bodyweight of between 6.5kg and 9kg. The x-axis depicts age in weeks, whereas the y-axis depicts bodyweight in kilograms.

**Fig 9:**
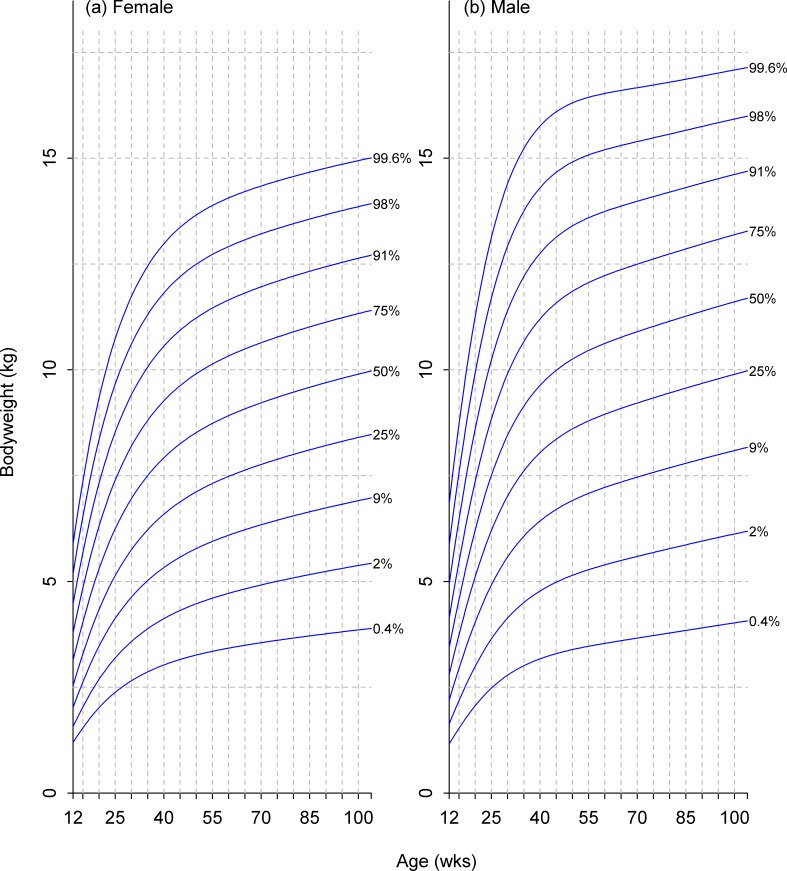
Growth standard chart for size category III. Left and right panels show the final curves for males and females, respectively, for dogs with a predicted adult bodyweight of between 9kg and 15kg. The x-axis depicts age in weeks, whereas the y-axis depicts bodyweight in kilograms.

**Fig 10:**
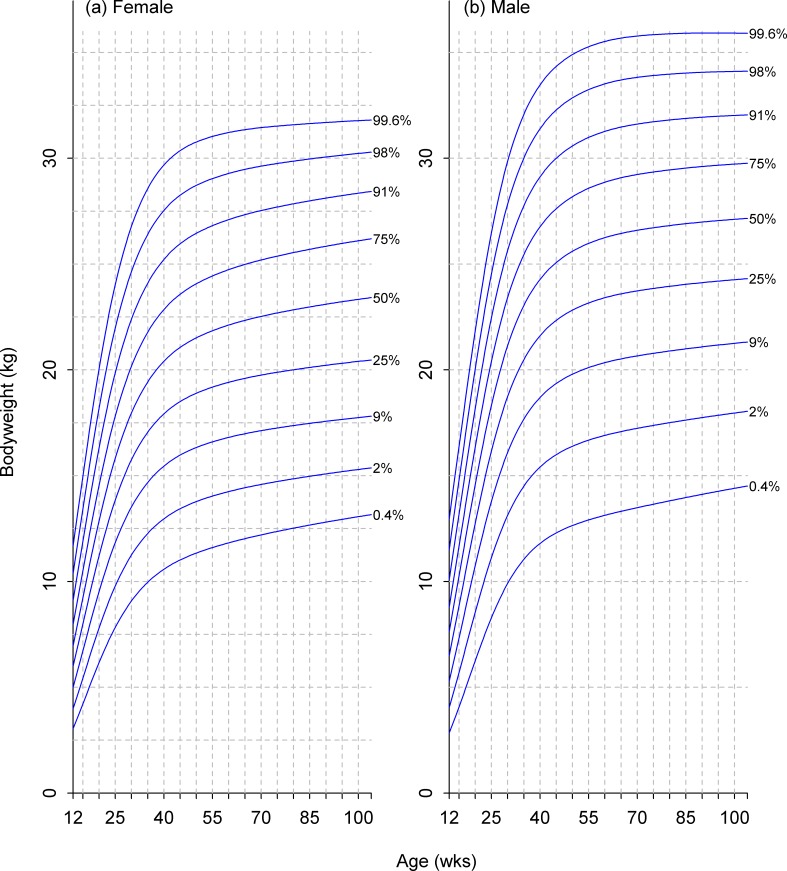
Growth standard chart for size category IV. Left and right panels show the final curves for males and females, respectively, for dogs with a predicted adult bodyweight of between 15kg and 30kg. The x-axis depicts age in weeks, whereas the y-axis depicts bodyweight in kilograms.

**Fig 11:**
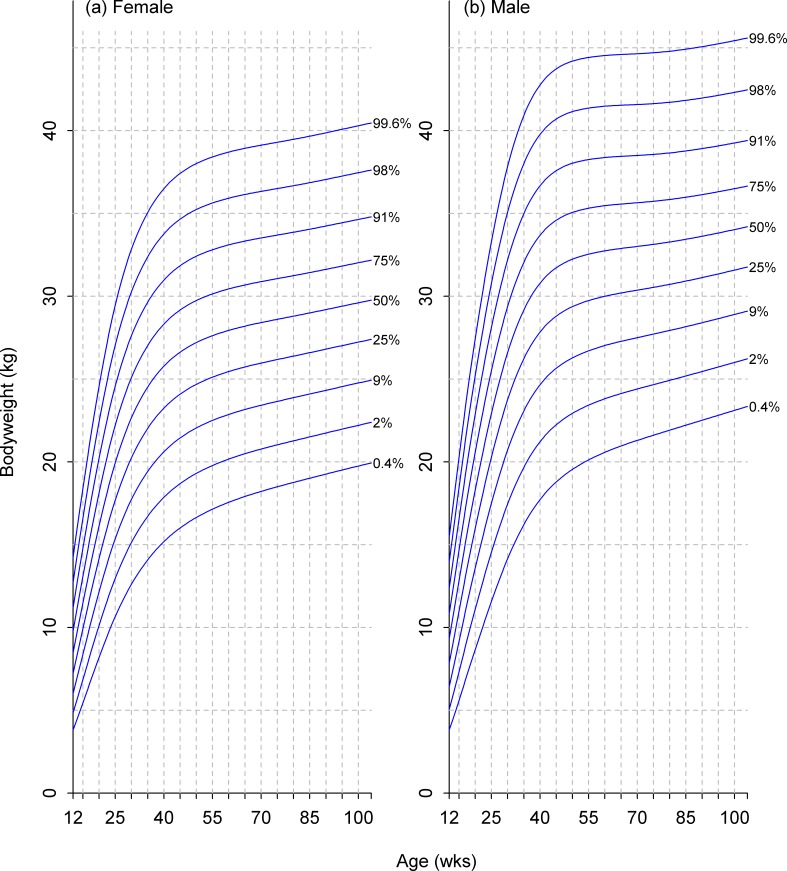
Growth standard chart for size category V. Left and right panels show the final curves for males and females, respectively, for dogs with a predicted adult bodyweight of between 30kg and 40kg. The x-axis depicts age in weeks, whereas the y-axis depicts bodyweight in kilograms.

Using a reference age of 6 months, predictions of the 24 remaining breed-specific growth curves (12 non-giant breeds × 2 sexes) were compared with those of the relevant size category. Ten of these showed a high level of consistency (not deviating by more than half the distance between neighbouring centiles at any time) whilst a further ten gave an acceptable level of consistency (i.e. none of the trajectories crossed neighbouring centile lines). Exceptions included Pug (male), Boston Terrier (male), Beagle (female) and the Pit Bull breed type (female), although the degree of centile crossing was not marked in any case. As a result, a decision was made to base growth curves for clinical use on the new breed size categorisations rather than on individual breeds. The final growth curves for male and female dogs of all five size categories are shown in Figs 7–11.

## Discussion

We have successfully mapped patterns of growth using age and bodyweight data from a large population of healthy pet dogs, and created growth centile charts that take account of the variability related to both breed and neutering. Whilst a single chart applicable to all sizes and shapes of dogs was never likely to be feasible, it was possible to create a limited number of growth charts that reflected growth across the majority of pet dogs in the database. After further validation, such charts could form the basis of a clinical tool to enable trained veterinary professionals to monitor growth objectively during early life. Such a tool would promote healthy growth and help veterinary professionals to identify individuals with possible growth disturbances. Further, if veterinary professionals can ensure that more dogs are in optimal body condition upon entering early adulthood, this should help to promote the maintenance of a healthy weight, through lifelong regular weight monitoring. Thus, whilst this would predominantly be a tool for growing dogs, it might have indirect benefits in promoting longer-term health for the whole dog population.

Charts are commonly used as an aid for monitoring growth of children, and are either 'growth references' or 'growth standards'. A growth reference describes the growth of a defined population but make no inference as to their health, whilst a growth standard describes the growth of ‘healthy’ infants, and is intended to represent an ideal [27]. An example of a growth reference is the UK 1990 growth charts, which reflected the growth of children in 1990 [28]. By contrast, the WHO Child Growth Standards described the growth of children living in a well-supported healthy environment in six different countries [2,3]. All infants were either exclusively or predominantly breastfed, raised in favourable socioeconomic conditions by mothers who followed WHO feeding recommendations and did not smoke, and were not subjected to problems likely to constrain growth. Whilst more challenging to develop, such a reference is preferable in that it demonstrates the characteristics of optimal growth in infants achieving their full genetic potential. The charts developed in the current study would best be described as a growth standard, as the eligible dogs were assumed to be healthy (e.g. by only using data used from dogs either attending routine vaccination visits or visits where the veterinarian selected 'healthy dog' as the diagnosis) and had remained in ideal body condition throughout the first 3 years of life.

The choice of statistical methods used in the current study were informed by those used for the WHO Child Growth Standards [2,3]. Both used GAMLSS modelling (specifically the BCPE model) and similar methods of judging model fit. However, this study differs in the methods of data cleaning and the necessity of investigating the potential influence of neutering and breed. Concerning the former, the WHO study excluded data considered to fall into an unhealthy weight-for-height range, whilst within-individual cleaning was undertaken manually by examination of each individual chart. In contrast, the current study used a different process to exclude data considered to be extreme in terms of weight-for-age, and supported this with a further stage of automated within-individual cleaning. These differences were necessary, firstly, because there was a lack of height (or equivalent) data, removing the possibility of examining weight-for-height and, secondly, because the resource was not available for complete examination of each individual dog’s chart.

Compared with modelling human growth, a particular challenge for modelling growth curves for dogs was the possible influence of neutering, which is an important risk factor for weight gain predisposing to overweight and obesity [17,29]. Given that both ovariohysterectomy and castration are commonly performed during the growth phase, it was necessary to examine their influence on growth both visually and quantitatively. Differences in growth pattern associated with neutering affected the centile curves, with earlier neutering (before 37wks of age) tending to cause upwards shifts and later neutering (after 37wks of age) downwards shifts in growth trajectory. Whether such shifts are the cause or the effect of age of neutering is not known. On the one hand, the hormonal effects of neutering could alter growth patterns; conversely, the pattern of growth might influence the timing of neutering, for instance, if neutering is delayed in an animal growing slowly to mitigate against possible risks associated with anaesthesia and surgery. Additional studies are required to determine the direction of causation. Although both visual examination and mathematical analyses demonstrated effects of neutering on growth at the population level, they were relatively small and were dwarfed by the variability seen amongst individual dogs. Of course, the neutering effect we observed might have been minimised by the fact that dogs were selected to have a healthy BCS in young adulthood. By excluding overweight dogs, we might either have eliminated those which experienced the greatest post-neutering effects, or have biased our selection towards dogs where post-neutering weight changes were corrected by changes in husbandry (for example reducing food intake when a rapid increase in weight was observed). Nonetheless, it suggests that, for dogs that remain healthy and in optimal body condition, the overall growth pattern is similar in neutered and intact dogs, and do not require separate growth standards.

We determined the effect of breed in two ways, firstly by creating breed-specific growth curves and then by creating curves based upon size categories. Whilst, arguably, breed-specific curves might more closely reflect the growth in dogs within each breed, they have the disadvantage of increased complexity given the number of charts required, limiting their utility by veterinary professionals in clinical practice. In addition to reducing the number of charts required, a size-category approach means the charts are suitable for many more breeds (i.e. not just those used to create them) and also to dogs of mixed breeding. However, one challenge faced was that, to the authors' knowledge, no standardised size grouping exists. Therefore, we initially chose to use an existing size classification [10] which, although not perfect, did at least provide a starting point for grouping breeds approximately on size, which could then be refined. However, initial modelling and subsequent hierarchical cluster analysis revealed problems with these original groupings. The first issues identified, namely that one grouping had a bimodal residual distribution, whilst others contained breeds which exercised excessive influence on the resulting model, were resolved by replacing the pre-existing classes with 6 new size categories. Hierarchical analysis on two larger sets of breeds was used to identify with more precision where the new class boundaries should be placed.

The hierarchical analysis also indicated possible problems with categorisation of the largest dog breeds. Firstly, the Rottweiler was originally within the large size class, but was substantially heavier than other breeds in this category. However, its growth pattern was also distinct from breeds in the giant size class. Further, the Great Dane and the Mastiff differed markedly from other breeds in the original giant size class. Visual inspection of breed-specific curves for these two breeds (which were the most common in their class) confirmed that a single curve of their average would not fit either. As a result, no further attempts at creating a unified set of curves for new size category VI were made. The difficulty we encountered is perhaps not unexpected, since a previous study also highlighted differences in growth pattern amongst giant breed dogs [10]. Therefore, further work is needed to create a series of breed-specific curves for individual giant breeds.

Nevertheless, growth curves for the five smaller size categories were successfully produced. Overall they agreed well with the corresponding breed-specific curves, and the occasional discrepancies appeared to result from inconsistencies in the breed-specific curve rather than the size curves. This suggests that an approach using size category curves would be valid for mapping growth trajectories for individual dog breeds, and potentially also for dogs of mixed breeding. Comparatively speaking, this observation is similar to the implementation of the WHO growth standards across a range of populations. These standards were developed from the WHO Multicentre Growth Reference Study [2,3], which utilised data from six different countries: Brazil, Ghana, India, Norway, Oman and USA, ensuring children were included from a variety of ethnic backgrounds and cultural settings [30]. Despite such differences, patterns of healthy growth were broadly similar, and these patterns are mirrored across subsequent studies conducted in other countries (Argentina, Italy, Maldives and Pakistan) [31]. Thus, as with size categories, healthy patterns of growth in humans are similar across different ethnicities. Therefore, we believe that the new size categories are appropriate for monitoring growth and constitute, for the first time, evidence-based size categories in terms of best fit from a physiological perspective.

As with any study, a number of limitations should be acknowledged. For example, the study was retrospective in nature, and utilised cross-sectional data collected over an extended period, from many locations, by many veterinary professionals. One advantage of this approach is that the available datasets were large and the results were representative of the pet dog population, living in a home environment, and derived from clinical information collected during ‘normal’ veterinary visits. As a result, the data are arguably more generalisable to pet dogs than would have been the case if a study had been undertaken in a research colony. However, a disadvantage of the 'natural variability' in the study population is that there is likely to be more background noise in the data. For instance, information provided by owners (e.g. date of birth and breed) might have been inaccurate, and errors might have been made in bodyweight measurements and in data inputting at the veterinary hospitals. Further, bodyweight measurements occurred more commonly at particular ages (e.g. coinciding with vaccinations and other health checks) such that data were not uniformly available across the entire growth period. In addition, a significant amount of data cleaning was undertaken, with exclusion of any data thought to be unreliable. Whilst such a cautious approach led to a significant loss of available data (Fig 1), the original datasets were large enough to accommodate this. One further consequence was that it was only possible to model changes in body weight, and no data were available to examine other growth changes such as length or height. Such data are not routinely collected in veterinary practice and therefore were not available for the study. That said, even if it were, the marked variability in size and shape across the canine population would been extremely challenging.

The growth curves were created to be representative of healthy growth by restricting the data to either routine vaccination visits or visits where 'healthy dog' was recorded as the diagnosis category. This disease category might have been selected in error, but we believe it to be unlikely. Dogs that were not in optimal body condition were also excluded, and it proved more difficult to ensure the accuracy of these assessments. Firstly, different systems were used to assess body condition during the period of data collection, with a 3-category assessment used initially, which was later replaced by a 5-point BCS system. To ensure consistency across the whole study period, 5-point scores were converted to the appropriate 3-category assessment and, whilst the conversion was straightforward (e.g. 5-BCS scores of overweight and markedly obese assigned the 'heavy' category, and 5-BCS scores of very thin and thin assigned the 'thin' category), errors might have been introduced as a result. A second issue regarding body condition was the fact that it was not mandatory for veterinarians to record it, possibly introducing reporting bias, whereby veterinarians would be more likely to record abnormal body condition than optimal body condition. Of greater concern was the fact that, if a veterinarian did not complete a score in the visit record, the computer system defaulted to a score of 'normal', rather than recording a null value. To exclude errors arising from this, a linear discriminant analysis was used to predict the most likely body condition category from bodyweight for the 33 most common breeds, with dogs excluded if their predictions were thought to be unreliable. These predictions were then used to identify dogs likely to be ‘normal’. In addition, the diagnosis category was cross-checked to identify cases where a diagnosis of underweight, overweight or obesity was received, and, where this conflicted with the body condition assessment, the latter was corrected. Despite these measures, we cannot be certain that the body condition assessments used were accurate, and we acknowledge this as a study limitation.

A third study limitation was the fact that all dogs were patients of a single corporate veterinary hospital network in the USA. Whilst the number of hospitals is large (over 900) and spread over a wide area, the patient population might not be fully representative of the diversity in different types of companion animal practice, for instance privately-owned veterinary hospitals, mixed practices, and those in rural areas. Further, since the population studied was from the USA, it might not be representative of pet dogs from other countries. Not only could genetic background be different, but there could likely be differences in climate, culture, socio-economic status, nutrition, and husbandry practices. Similar to the work undertaken for the WHO growth standards [2,3], additional studies using other populations of dogs should be used to validate the growth charts. A final issue regarding the data used was the fact that they were collected over a period of 20 years. During this time, many changes would be expected in veterinary practice protocols, expertise, technology, and data recording. Further, the prevalence of diseases will have changed, as well as awareness of them. That said, as a result of growth in Banfield Pet Hospital clientele, the majority of the data were gathered from the second 10-year period, which reduced the magnitude of any timeframe effect. This is also a study limitation, and further validation with newer datasets should be considered.

## Conclusions

A series of evidence-based growth standards, based on bodyweight, have been developed for male and female dogs across 5 different size categories. Although the standards are valid for most dogs, they do not apply dogs with an adult weight >40 kg. The effects of neutering on the growth pattern were small with respect to the variability seen amongst individuals and alterations to the growth standards were not needed. Further work is now required to validate these growth standards and provide training to veterinary professionals, so that they can be used as a clinical tool for objective monitoring of growth in pet dogs. Work is also required to develop standards for dogs >40kg.

## Supporting information

S1 Table Checklist for the STROBE and RECORD statements. The table lists the items of the respective checklist, and the location within the manuscript where they can be found.
(PDF)
