# Correction: Growth standard charts for monitoring bodyweight in dogs of different sizes

**DOI:** 10.1371/journal.pone.0314711

**Published:** 2024-11-26

**Authors:** Carina Salt, Penelope J. Morris, Alexander J. German, Derek Wilson, Elizabeth M. Lund, Tim J. Cole, Richard F. Butterwick

The Data Availability statement for this paper is incorrect. The correct statement is: The study data are available from the Liverpool Data catalogue (https://doi.org/10.17638/datacat.liverpool.ac.uk/377). Please note that the dataset has been fully anonymised by removing any client and animal details that might enable the client to be identified.
